# Effects of Exercise Training during Christmas on Body Weight and Cardiometabolic Health in Overweight Individuals

**DOI:** 10.3390/ijerph17134732

**Published:** 2020-07-01

**Authors:** Miguel Ramirez-Jimenez, Felix Morales-Palomo, Juan Fernando Ortega, Alfonso Moreno-Cabañas, Valle Guio de Prada, Laura Alvarez-Jimenez, Ricardo Mora-Rodriguez

**Affiliations:** 1 Exercise Physiology Lab at Toledo, University of Castilla-La Mancha, 45071 Toledo, Spain; miguel.ramirez@uclm.es (M.R.-J.); felix.morales@uclm.es (F.M.-P.); juanfernando.ortega@uclm.es (J.F.O.); Alfonso.Moreno@uclm.es (A.M.-C.); MValle.Guio@alu.uclm.es (V.G.d.P.); laura.alvarez@uclm.es (L.A.-J.); 2 Sports Medicine Center, Diputacion de Toledo, 45002 Toledo, Spain

**Keywords:** detraining, weight gain, dyslipidemia, insulin sensitivity, holidays, cardiovascular diseases

## Abstract

Individuals with abdominal obesity and metabolic syndrome (MetS) have augmented risk of all-cause mortality. Lifestyle interventions are effective to treat MetS, however, there are periods during the year in which exercise programs are discontinued and improper dietary habits reappear (e.g., Christmas holidays). We aimed to analyze if exercise-training during Christmas holidays would avoid body-weight gains and cardiometabolic deterioration in MetS individuals, using a randomized control trial. Thirty-eight men with MetS undergoing exercise training were randomly allocated to either continue (TRAIN group, n = 16) or discontinue (HOLID group, n = 22) training, during the three weeks of Christmas. Anthropometrics (body weight, fat, and waist circumference), fasting blood metabolites (glucose, insulin, triglycerides, and cholesterol concentrations) and exercise maximal fat oxidation (FO_MAX_) and oxygen uptake (VO_2PEAK_) were determined before and after Christmas. Both groups were similar at baseline in all parameters (*p* > 0.05). HOLID group increased body weight (91.3 ± 13.0 to 92.0 ± 13.4 kg, *p* = 0.004), mean arterial pressure (94.0 ± 10.6 to 97.1 ± 8.9 mmHg, *p* = 0.026), blood insulin (10.2 ± 3.8 to 12.5 ± 5.4 µIU·mL^−1^, *p* = 0.003) and HOMA (3.2 ± 1.3 to 4.1 ± 2.3, *p* = 0.003). In contrast, TRAIN prevented those disarrangements and reduced total (170.6 ± 30.6 to 161.3 ± 31.3 mg·dL^−1^, *p* = 0.026) and low-density lipoprotein cholesterol (i.e., LDL-_C_, 104.8 ± 26.1 to 95.6 ± 21.7 mg·dL^−1^, *p* = 0.013). TRAIN also prevented the reductions in exercise FO_MAX_ and VO_2PEAK_ that was observed in the HOLID group (*p* = 0.002). In conclusion, exercise training during Christmas, prevents body weight gains and the associated cardiovascular (increase in blood pressure and LDL_-C_) and metabolic (reduced insulin sensitivity) health risks are an optimal non-pharmacological therapy for that period of the year.

## 1. Introduction

Metabolic syndrome (MetS) is a cluster of cardiovascular and metabolic derangements that increases 2-fold the risk of cardiovascular mortality and 1.5-fold the risk of all-cause mortality [1]. MetS factors include elevated blood pressure, dyslipidemia (raised triglycerides and lowered high-density lipoprotein cholesterol), fasting glucose, and waist circumference, all of which are related to weight gain [2]. Waist circumference, a surrogate of intra-abdominal fat accumulation, is one of the most frequent factors of the MetS, with an increasing prevalence over the past decades [3]. Obesity is considered the central feature of the MetS, preceding the appearance of insulin resistance, which subsequently leads to elevation in the remaining factors [4]. It has been proposed that the excess of energy that occurs in weight gain leads to an energy surplus, with accumulation of intramuscular lipid intermediaries, inducing insulin resistance [5]. Therefore, weight gain and obesity should be a target to prevent and treat MetS.

Lifestyle modifications remain the first-line therapy for patients with MetS. Both diet and exercise are advised to create a negative energy balance that could reduce obesity. In addition, aerobic exercise training enhances cardiorespiratory fitness (CRF), which is negatively associated with MetS prevalence and the risk of suffering cardiovascular disease and all-cause mortality [6]. Furthermore, superior benefits on CRF and further improvements on MetS components are sometimes obtained from high-intensity interval training (HIIT), in comparison to moderate intensity continuous training [7]. However, other studies suggest that training volume is a greater determinant than exercise intensity for improving MetS [8,9]. Nevertheless, HIIT is a time-efficient training type, which could help individuals with busy schedules to engage in exercise programs.

Although lifestyle interventions are effective to treat MetS [6,7], there are periods during the year in which exercise programs are discontinued and improper dietary habits reappear (e.g., Christmas holidays). Particularly during Christmas, energy intake increases in combination with reduced energy expenditure from physical activity [10,11]. As a result, it was reported that more than half of the increases in body weight during adulthood take place during Christmas holidays [12]. Other studies have confirmed Christmas as a period of weight gain in industrialized societies (i.e., Japan, United States, Germany [13]), however, the effects of this weight gain on cardiometabolic health variables is not usually reported. To date, only one observational study has addressed the effects of self-reported physical activity on body weight and health-related parameters, without finding any protective effect [14]. Therefore, to our knowledge, no study has investigated if a life-style intervention during this key period of the year would prevent weight gain and the worsening of MetS components. 

The purpose of this study was to assess the effects of exercise training during 3 weeks of Christmas holidays (20 December–10 January) on body weight, cardiometabolic health parameters, and fat oxidation capacity, in a sample of MetS individuals. We used randomized control trial, where two groups of well-matched individuals (based on presence of MetS components) were either exercise-trained or not, during Christmas. Our hypothesis was that exercise-training during Christmas holidays would avoid body weight gains and cardiometabolic deterioration in MetS individuals.

## 2. Methods

### 2.1. Participants 

Thirty-eight middle-aged (57 ± 8 years) men with overweight (BMI 32 ± 5 kg·m^−2^) and metabolic syndrome, undergoing a three-months cycling high-intensity interval training (HIIT) program completed this study. Metabolic syndrome was defined as the presence of three of the following five risk factors; elevated waist circumference, blood pressure, fasting blood glucose, triglycerides, or reduced high density lipoprotein-cholesterol (HDL-_C_) [2]. Exclusion criteria included untreated cardiovascular disease, or any condition associated with exercise intolerance. All subjects provided written, witnessed, informed consent of the protocol approved by the local Hospital’s Ethics Committee (reference #170), in accordance with the World Medical Association Declaration of Helsinki. ClinicalTrials.gov identifier: NCT03019796.

### 2.2. Experimental Design 

**Figure 1:**
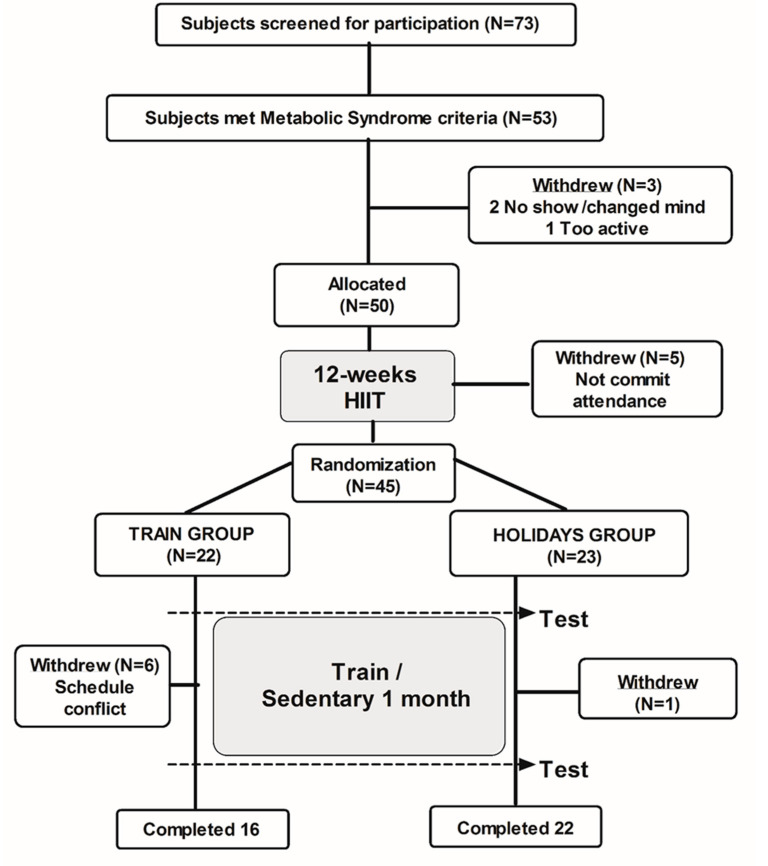
CONSORT schematic representation of the study procedures.

Volunteers were recruited, clinically screened, and randomized, as presented in Figure 1. One week before Christmas, all participants were tested for baseline assessment, which coincided with the end of their training program. Following this, participants were allocated to either the TRAIN group (n = 16) or the HOLID group (n = 22), using a blinded, randomized (stratified by number of MetS factors) block-controlled design. The TRAIN group remained in training, whilst the HOLID group interrupted their supervised training schedule. All subjects were advised to maintain their dietary habits and physical activity patterns during the intervention, in order to preserve the ecological design of this study. We used recall questionnaires (7-day IPAQ [15] and 3-day nutritional diary CESNID v1.0 [16]; Barcelona, Spain) at the end of the intervention, to estimate those two important variables (i.e., physical activity and caloric intake), during Christmas.

Training consisted of pedaling on a stationary bicycle for 10-min, as a warm-up set at an intensity of 70% of individuals’ maximal heart rate (i.e., HR_MAX_). This was followed by 4 × 4-min intervals at 90% of HR_MAX_, interspersed with 3-min of active recovery at 70% HR_MAX_, to end with a 5-min cool-down period, for a total of 43 min workout. During the 3-week study period, subjects completed nine of these training sessions (3 per week) and could miss a maximal of 1 session to be included in the study. In TRAIN, the tests were conducted at least 48-h after the last exercise training session, to avoid testing the acute effects of the last exercise bout.

### 2.3. Clinical Investigation 

Subjects arrived at the laboratory in the morning, after an overnight fast (≥8 h). Nude body weight (Hawk, Metler, Toledo, USA), height (Stadiometer, Secca 217, Hamburg, DE), waist circumference (2 cm above the iliac crest), and body composition by bioimpedance (Tanita BC-418; Tanita Corp, Tokyo, Japan) were assessed by the same researcher. Then, the resting blood pressure was measured after 15 min of supine resting, using a calibrated ECG-gated electro-sphygmomanometer (Tango, SunTech Medical; NC; USA) as the average of three measurements. Mean arterial pressure (MAP) was calculated as follows:

MAP = 2/3 diastolic blood pressure (DBP) + 1/3 systolic blood pressure (SBP).

Following this, a 5-cc blood sample was drawn from an antecubital vein for the determination of blood glucose, insulin, and blood lipid profile (i.e., triglycerides, total cholesterol, and HDL-_C_).

### 2.4. Cardio-Respiratory and Metabolic Fitness Assessment 

Next, maximal fat oxidation (FO_MAx_) was assessed after an overnight fast on an electronically braked cycle ergometer (Ergoselect 200, Ergoline, Germany), using a submaximal graded exercise test (GXT). Exhaled air was continuously collected and analyzed breath-by-breath for oxygen consumption and carbon dioxide production, using indirect calorimetry (Quark b2; Cosmed, Italy). Initial power-output was set at 30 W and, every 4 min, the power-output increased 15 W, until the respiratory exchange ratio (RER = VCO_2_/VO_2_) exceeded 1.0. The last minute of each stage was averaged to calculate the non-protein respiratory quotient and fat oxidation rate [17]. After 15 min of passive rest and rehydration (200 mL of a fruit juice with 20 g carbohydrate), the peak aerobic capacity (VO_2PEAK_) was assessed during a maximal GXT, until volitional exhaustion with 12-lead ECG (Quark T12, Cosmed, Italy), to ensure normal heart function. After 3 min of warm-up at 50 W, power-output increased every minute by 20 W, until volitional exhaustion. In cases of absence of oxygen consumption (VO_2_) plateau, the secondary criteria (RPE ≥ 17; RER > 1.1, and an HR > 85% maximal predicted) were considered. The highest VO_2_ obtained during the test was considered as the VO_2PEAK_.

### 2.5. Blood Analyses and Insulin Sensitivity 

Plasma glucose was analyzed using the glucose oxidase peroxidase method with an intra–inter assay coefficient of variation (iCV) of 0.9 ± 1.2%. Insulin concentration was measured in duplicates, using chemiluminescent micro particle immunoassay (iCV; 2.0–2.8%) in an automated immunoassay analyzer (Architect ci4100, Abbott Laboratories, USA). Insulin sensitivity was calculated using the homeostasis model assessment of insulin resistance (HOMA-IR), as follows:

HOMA-IR = (Fasting Plasma Glucose × Fasting Plasma Insulin)/22.5

HDL-c was measured using the accelerator selective detergent method (iCV; 1.7 ± 2.9%). Blood triglycerides (TG) was measured with glycerol-3-phosphate oxidize method (iCV; 0.8 ± 1.7%). Total serum cholesterol (TChol) was measured by an enzymatic method with a single aqueous reagent (iCV; 1.1 ± 1.4%). Low-density lipoprotein-cholesterol (LDL-c) was calculated as proposed by Friedewald [18], as follows:

LDL-c = TChol − HDL-c − (TG/5)

All of the above analyses were run in an automated Mindray BS 400 Chemistry Analyzer (Mindray Medical Instrumentation, Shenzhen, China).

### 2.6. Statistical Analysis 

Data are presented as mean ± SD, unless otherwise indicated. Sample size was calculated, based on one of our primary outcome measurements (body weight), from a database of 134 participants who finished a 16-weeks aerobic training program. Power test revealed that at least 21 subjects were needed to detect changes of 0.5 ± 0.9 kg in body weight, to reach a significance for a statistical power at 80% (α = 0.05). All variables showed a normal distribution according to the Shapiro–Wilks test. Intergroup differences at baseline were tested by unpaired Student *t*-test. Mixed-design analysis of variance (ANOVA) was used to compare differences across time (baseline vs. 3-weeks) and groups (TRAIN vs. HOLID) in all variables. When the time–group interaction was significant, Bonferroni post-hoc testing was performed to detect changes within groups. Associations between pre-post changes in selected variables were explored using Pearson correlation coefficient (r). Statistical analysis was performed using SPSS, v22 (IBM Corporation, Armonk, New York, USA).

## 3. Results

### 3.1. Caloric Intake, Physical Activity, and Mets Factors

Thirty-eight participants completed the experiment, 16 in the TRAIN and 22 in the HOLID group. Analysis of the recall questionnaires revealed no differences in calorie intake or physical activity between groups. On average, subjects ingested 2596 ± 93 kcal·day^−1^ in the last three days of the experiment. Macronutrient distribution in both groups was also similar (47 ± 5% carbohydrate, 33 ± 2% fat [50% saturated fat] and 20 ± 1% protein). On average, for both groups, the 7-days recall IPAQ revealed physical activity during the Christmas of 1810 ± 1524 MET-min/week.

Participants were similar in all metabolic syndrome components at baseline (*p* > 0.05; Table 1). Systolic and diastolic blood pressure increased significantly in HOLID (e.g., 127.6 ± 14.3 to 131.3 ± 13.1 mmHg for systolic, *p* = 0.029), whereas there were statistically insignificant decreases in the TRAIN group (Table 1). Neither TRAIN nor HOLID significantly affected the fasting blood glucose, HDL-_C_, or triglycerides (Table 1). HOLID waist circumference increased (108.1 ± 10.3 to 110.1 ± 9.4 cm, *p* = 0.002), while increases in the TRAIN group were not statistically significant (111.9 ± 11.7 to 112.2 ± 12.1 cm, *p* = 0.678).

### 3.2. Body Composition, Carbohydrate, and Lipid Metabolism 

**Figure 2:**
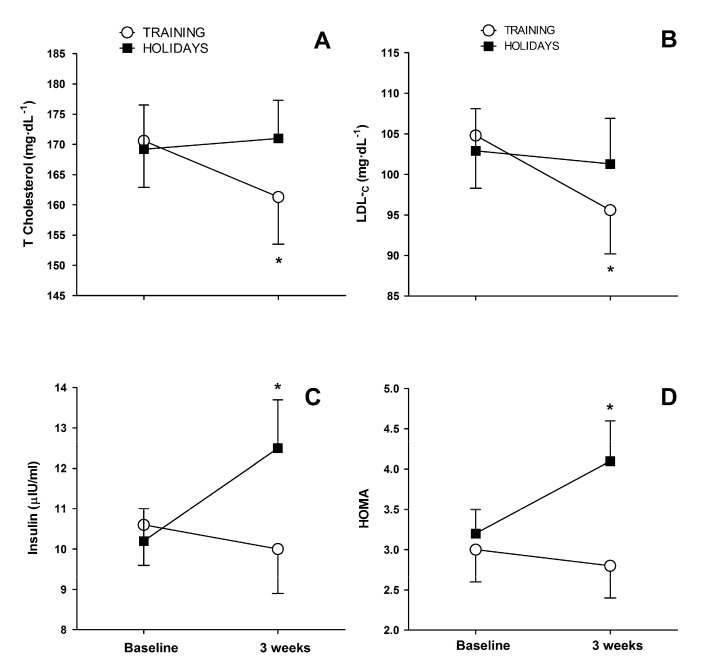
Panel (**A**) (total cholesterol) and (**B**) (LDL-_C_) represent lipid metabolism, whilst panel (**C**) (insulin) and (**D**) (HOMA) represent carbohydrate metabolism, before and after 3-weeks of Christmas in the TRAIN and HOLID groups. Values are means ± SEM. * Significant difference between baseline and three weeks for that group (*p* < 0.05).

Calculated body fat changes in groups were not statistically significant: HOLID group (28.6 ± 7.2 to 28.8 ± 7.7 kg, *p* = 0.569) and TRAIN group (32.9 ± 11.2 to 32.2 ± 11.6 kg, *p* = 0.132). Total cholesterol (170.6 ± 30.6 to 161.3 ± 31.3 mg·dL^−1^, *p* = 0.026) and LDL-_C_ (104.8 ± 26.1 to 95.6 ± 21.7 mg·dL^−1^, *p* = 0.013) decreased in the TRAIN group. However, in the HOLID group, the total cholesterol (169.2 ± 34.4 to 171.0 ± 29.8 mg·dL^−1^, *p* = 0.616) and LDL-_C_ (102.9 ± 24.6 to 101.3 ± 26.2 mg·dL^−1^, *p* = 0.576) did not change significantly from baseline (Figure 2A,B). Fasting insulin (10.2 ± 3.8 to 12.5 ± 5.4 µIU·mL^−1^, *p* = 0.003) and HOMA-IR (3.2 ± 1.3 to 4.1 ± 2.3, *p* = 0.003) increased significantly after Christmas in the HOLID group. No significant changes in fasting insulin (10.6 ± 4.1 to 10.0 ± 4.4 µIU·mL^−1^, *p* = 0.562) or HOMA-IR (3.0 ± 1.5 to 2.8 ± 1.5, *p* = 0.602) were observed in the TRAIN group (Figure 2C,D).

### 3.3. Cardiorespiratory and Metabolic Fitness Parameters 

**Figure 3:**
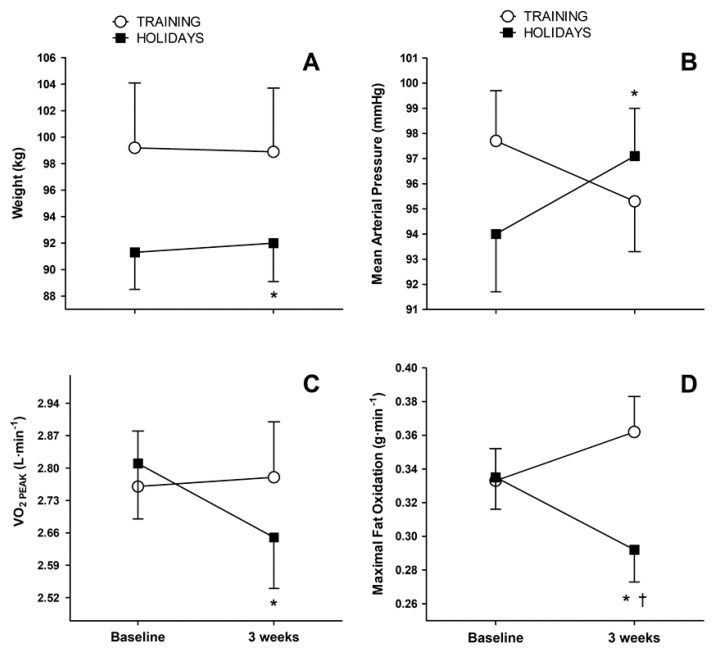
Body weight (panel (**A**)), mean arterial pressure (panel (**B**)), and exercise parameters (VO_2PEAK_ and FO_MAX_) in (panel (**C**) and (**D**)) before and after 3-weeks of Christmas in the TRAIN and HOLID groups. Values are means ± SEM. * Significant difference between baseline and 3 weeks, for each group (*p* < 0.05). † Significant difference between groups at the specified time-point (*p* < 0.05).

Body weight increased from 91.3 ± 13.0 to 92.0 ± 13.4 kg (*p* = 0.004) in the HOLID group, while in the TRAIN group, body weight decrease from the baseline values were not statistically significant. (99.2 ± 19.6 to 98.9 ± 19.3 kg; *p* = 0.371, Figure 3A). Mean arterial pressure increased in the HOLID (94.0 ± 10.6 to 97.1 ± 8.9 mmHg, *p* = 0.026), while in the TRAIN group, the decrease from baseline was not statistically significant (97.7 ± 7.9 to 95.3 ± 8.2 mmHg, *p* = 0.116, Figure 3B). Three-weeks of detraining decreased VO_2PEAK_ (2.81 ± 0.54 to 2.65 ± 0.53 L·min^−1^, *p* = 0.002) in the HOLID, while the TRAIN group maintained VO_2PEAK_ at baseline levels (2.76 ± 0.48 to 2.78 ± 2.49, *p* = 0.665; Figure 3C). Maximal fat oxidation (FO_MAX_) was non-significantly increased in the TRAIN (0.33 ± 0.08 to 0.36 ± 0.08 g·min^−1^, *p* = 0.052), while it decreased in the HOLID (0.33 ± 0.09 to 0.29 ± 0.09 g·min^−1^, *p* = 0.002; Figure 3D).

### 3.4. Correlation Analysis 

The changes between baseline and 3 weeks of Christmas for the HOLID group in several responses were correlated (Table 2). Body weight gain correlated with increases in LDL-_C_ (r = 0.560; *p* = 0.007), systolic blood pressure (r = 0.419; *p* = 0.052), and reductions in exercise maximal fat oxidation (FO_MAX_, r = 0.679; *p* = 0.001). In turn, the reductions in FO_MAX_ correlated with increases in systolic blood pressure (r = 0.477; *p* = 0.025). Likewise, the increases in LDL-_C_ correlated with increases in systolic blood pressure (r = 0.494; *p* = 0.019). Increases in fasting blood triglycerides correlated with worsening of insulin sensitivity (i.e., HOMA-IR, r = 0.425; *p* = 0.048).

## 4. Discussion

To our knowledge, this is the first study that observed if exercise training prevented holiday-related weight-gain and worsening of metabolic syndrome (MetS). We studied overweight and obese (i.e., BMI 31.8 ± 5.2 cm·kg^−2^) MetS individuals, because weight-gain rapidly worsened their cardio metabolic health [19] and, thus, prevention measures were clinically relevant. The main finding from our study was that exercise training during 3-weeks of Christmas prevented weight gain in the TRAIN group. In contrast, the HOLID gained weight (0.67 ± 1.03 kg; *p* = 0.004), while blood pressure, waist circumference, insulin sensitivity, cardiorespiratory fitness (i.e., measured as VO_2PEAK_), and the capacity to oxidize fat during exercise deteriorated. Body weight increased and the related disturbances were avoided with only nine bouts of training, during the three weeks of Christmas. Thus, our data suggest that a supervised exercise program during Christmas is an efficient strategy to avoid body weight gain and its associated deleterious effects, on the health of MetS individuals.

Yanovski and colleagues first assessed the real magnitude of seasonal increase in body weight between mid-November and mid-January, in a group of 195 racially mixed individuals, with a wide range of ages and BMI. The authors found 0.37 ± 1.52 kg of body weight increase, which were not reversed during the following months, contributing to 51% of the yearly weight gain [12]. Furthermore, obese people displayed larger holiday weight gain, suggesting that those individuals might benefit more from efforts to prevent weight gain. Questionnaires revealed that both increased hunger and reduced physical activity during holidays correlated with weight gain. Although the authors could not determine which of these factors was responsible for weight gain, they hypothesized that increased physical activity could prevent holiday-related weight gain in overweight people. Almost two decades after this seminal paper, we can confirm that their hypothesis about overweight and obese MetS individuals was correct. Of note, exercise prevented weight gain during Christmas (Figure 3A), despite self-reported large and similar caloric intake in both groups (2596 ± 93 kcal·day^−1^).

Hull and coworkers reported weight gains of 0.8 kg in overweight or obese college students, mostly due to gains in fat mass during Christmas [20]. In our study, we found 0.67 kg weight gain in the HOLID group, which correlated with increases in waist circumference, which was our proxy of abdominal fat. However, whole body fat content did not significantly increase in the HOLID or significantly decrease in the TRAIN. Accounting for the difference between our results and Hull’s, body composition changes in our study were measured by bioelectrical impedance, which is less precise than the DXA technology used in Hull’s study. An observational study by Cook et al. [21] explored if baseline energy expenditure (measured by doubly labeled water) could protect against weight gain during holidays. However, neither a high level of baseline energy expenditure [21] nor self-reported moderate physical activity [14] was protective against holiday weight gain. In contrast, we present results showing that when exercise was metered (duration and intensity) and supervised, it compensated for the likely increase in energy intake during Christmas, avoiding weight gain. We recently observed that body weight loss during an exercise training intervention was the stronger predictor of MetS evolution [22]. Currently, we found that avoiding holiday weight gain through exercise interventions prevented MetS from worsening during Christmas.

Impaired lipid metabolism is common in insulin-resistant states, such as the metabolic syndrome [19]. Aerobic exercise improves atherogenic dyslipidemia (i.e., elevated triglycerides, LDL_-C_, and reduced HDL_-C_), with better results from higher volumes of training, rather than from higher training intensities [23]. Although the beneficial effects of regular exercise on lipids and lipoproteins are well-documented [24], total cholesterol, and LDL-_C_ have shown poor responsiveness to short-term exercise training interventions [25]. In contrast, we currently report that TRAIN lowered total cholesterol and LDL-_C_, despite the environment of overeating during the Christmas holidays. On the other hand, the sedentary group in our study did not show a worsening of their blood lipid profile despite gaining 0.67 ± 1.03 kg. In a well-designed study by Walhin et al. [26], one-week of overfeeding and inactivity was enough to increase total blood cholesterol and LDL-_C_. However, in that week, body weight increased 2.8 kg, suggesting that large increases in body weight are needed to raise blood lipids. Previous data from our laboratory revealed that when body weight is maintained in overweight young adults, short-term exercise training (i.e., 11 sessions within 2 weeks) counteracts the effects of raising dietary saturated fat on blood LDL-c and total cholesterol [27]. We currently report that exercise can even lower those levels (i.e., Figure 2A,B). Blood levels of total cholesterol and LDL-_C_ are of great clinical interest, given that LDL-c is strongly linked to cardiovascular risk [24]. Therefore, the improvement in blood lipid profile in TRAIN is particularly relevant during a period of the year (i.e., Christmas), characterized by a deterioration in cardiometabolic profile.

Insulin resistance has a key role in the pathogenesis of the metabolic syndrome [19]. A bout of exercise has an acute effect on improving insulin sensitivity, which however, disappears 48 h after exercise [28]. Aerobic exercise training of enough duration (i.e., months) chronically improves insulin action, mainly by enhancing the peripheral tissue insulin sensitivity [29]. Recently, we have reported that insulin sensitivity is improved by aerobic training, only when body weight loss is achieved [30]. Accordingly, we currently report maintenance of insulin sensitivity (assessed by HOMA-IR) in the TRAIN group, which did not gain any weight through exercise training (Figure 2C). Thus, it seems that, in TRAIN, energy expenditure from exercise compensated for the increased Christmas calorie intake, preserving body weight, which is directly linked to insulin sensitivity [30]. We also reported that HOMA reverts to pre-training levels when exercise ceases by one-month, in MetS individuals [16]. Here, we observed that 3 weeks of detraining also deteriorated insulin sensitivity, mostly by increasing fasting blood insulin concentration (Figure 2C). Our data evidenced that Christmas holidays is a critical season for carbohydrate metabolism, because it combines frequent periods of overeating with reduced levels of physical activity, both being independent factors that impair insulin sensitivity [31,32].

Overeating, emotional stress, and reduced physical activity are factors related to increased heart failure events during Christmas holidays [33]. Hypertension is the most common recurring factor in MetS [34] and an independent risk factor for cardiovascular diseases and cardiovascular mortality. One of the key factors underlying the pathogenesis of hypertension is obesity [34], and weight gain increases the odds for becoming hypertensive [35]. It has been reported that a quick increase of body weight gain in Christmas of 0.78 kg is associated with increases in SBP and DBP of 2.3 and 1.8 mmHg, respectively [14]. We showed a similar increase (i.e., 3 mmHg in mean arterial pressure; Figure 3B) in the group that ceased training during Christmas (HOLID). In this group, the worsening of systolic blood pressure was correlated with the increases in body weight and LDL_-C_ (Table 2). This finding highlights the importance of body weight on blood pressure control, both variables being positively correlated. We reported that in obese MetS individuals, 4 months of exercise training lowered systolic blood pressure by 12%. Furthermore, 1 month of detraining without weight gain, did not return blood pressure to the levels prior to the onset of training [16]. The current findings suggest that even in a short period of time (i.e., 3 weeks) the gains in body weight negatively affect blood pressure and conversely maintain body weight (either by exercise or diet [36]), which is key to the prevention of developing hypertension.

Cardiorespiratory fitness (CRF) is inversely associated with the incidence of MetS and is a significant predictor of all-cause and cardiovascular mortality [6,37]. To enhance CRF, systematic and regular endurance training is required during several weeks. However, a few days of inactivity are enough to lose most part of the achieved benefits. In our group of individuals diagnosed with MetS, 3-weeks of detraining after holidays decreased VO_2PEAK_ by 5.6% (Figure 3C). Our group reported similar findings in MetS patients after 1 month of detraining, after 16-weeks of HIIT [16]. Detraining during Christmas blunted a surrogate measurement of mitochondrial oxidative capacity (i.e., FO_MAX_) by 13% (Figure 3D). Furthermore, our correlations indicate that body weight gains in the HOLID group was strongly associated with the deterioration of FO_MAX_ (Table 2). Conversely, the TRAIN group enhanced FO_MAX_ during Christmas. FO_MAX_ improvement after training was related to increased lipid mobilization and oxidation [38]. This enhancement in fat oxidation might be behind the reductions in blood concentrations of both LDL-_C_ and total cholesterol observed in the training group.

This study is not free of limitations. We only tested Caucasian male, MetS individuals, and thus, the results are only applicable to this population. We did not measure caloric intake or physical activity during the 3 weeks of Christmas and recall questionnaires were used to collect those data. Thus, caloric intake underreporting might have occurred. On average, caloric expenditure of the training bouts exceeded 400 kcals [39], which might not be feasible for all individuals. It is uncertain if a lesser dose of exercise would accomplish similar results. Finally, a difference (non-significant) between groups in baseline body weight was apparent (Figure 3A). We randomized-blocked subjects based on the number of MetS factors and not body weight, and fortuitously a 154 kg subject (i.e., 343 pounds) was ascribed to the TRAIN group while the average body weight when that subject was taken away was 95 kg (211 pounds). However, the analysis of the responses of this subject was similar to the rest of the group and, thus, we found no reason to exclude his data.

## 5. Conclusions

In conclusion, the increased risk for type 2 diabetes and cardiovascular disease in individuals with metabolic syndrome [1], demands special therapeutic attention in this population. The fundamental life-style therapeutic approach for this population is to reduce caloric intake to induce weight reduction and to increase physical activity. However, during Christmas holidays, intents of reduced caloric intake collide with a sociocultural environment promoting calorie-rich food consumption. Given that it is hard to avoid overeating during Christmas, we investigated if exercise training could prevent weight gain during this period. Of novelty, we measured an array of variables related to cardiometabolic health to document the effects of Christmas weight gain/maintenance on health. Furthermore, using a randomized control trial experimental design, we studied a sample of MetS individuals because weight gain rapidly worsened their cardiometabolic health, and thus, prevention measures are clinically relevant. Against what could be inferred from observational studies [14,21], and according to the hypothesis laid out in the seminal weigh-gain study of Yanovski et al. [12], we found that exercise training prevented weight gain and the associated cardiometabolic disarrangements (increased blood pressure, LDL-c, and reduce insulin sensitivity) in a background of increased caloric intake. Our results point to exercise as a practical non-pharmacological therapy, to prevent health deterioration during Christmas.

## Figures and Tables

**Table 1:** Evolution of the metabolic syndrome factors and body weight in the TRAIN and HOLID groups, during Christmas.

	TRAIN (n = 16)		HOLID (n = 22)		TRAIN vs. HOLID at Baseline	Time × Group
	Baseline	3 Weeks	Baseline	3 Weeks	*p* Value	*p* Value
Systolic blood pressure (mmHg)	131.3 ± 11.6	128.3 ± 11.1	127.6 ± 14.3	131.3 ± 13.1 *	0. 387	0.010
Diastolic blood pressure (mmHg)	80.9 ± 6.9	78.8 ± 7.4	77.2 ± 9.5	80.0 ± 7.8 *	0.179	0.013
Mean arterial pressure (mmHg)	97.7 ± 7.9	95.3 ± 8.2	94.0 ± 10.6	97.1 ± 8.9 *	0.230	0.010
Glucose (mg·dL^−1^)	111.1 ± 24.6	109.5 ± 19.5	125.0 ± 30.9	126.7 ± 34.3	0.144	0.546
HDL-_C_ (mg·dL^−1^)	41.4 ± 8.1	39.9 ± 9.2	44.1 ± 12.4	45.7 ± 12.9	0.422	0.215
Triglycerides (mg·dL^−1^)	122.0 ± 37.1	128.5 ± 60.4	111.0 ± 39.6	119.8 ± 39.7	0.385	0.880
Waist Circumference (cm)	111.9 ± 11.7	112.2 ± 12.1	108.1 ± 10.3	110.1 ± 9.4	0.309	0.079
Body weight (kg)	99.2 ± 19.6	98.9 ± 19.3	91.3 ± 13.0	92.0 ± 13.4 *	0.177	0.011

Data are presented as mean ± SD for 38 metabolic syndrome patients, divided into the TRAIN (n = 16) and HOLID (n = 22) groups. * Significantly different from baseline within that group. To convert HDL-c, glucose, and triglycerides to mmol·L^−1^, multiply by 0.0259, 0.0555, and 0.0113, respectively.

**Table 2:** Correlations (Pearson r) between selected changes (∆ Pre–Post Christmas holidays) in cardiovascular, metabolic, and anthropometric variables in the HOLID group.

LDL-_C._	Triglycerides	Body Weight	FO_MAX_	VO_2PEAK_	
−0.096	0.425 *	0.036	−0.262	−0.183	**HOMA**
0.494 *	−0.138	0.419	−0.477 *	−0.290	**SBP**
0.032	0.494 *	0.181	−0.199	0.179	**Resting HR**
	−0.468 *	0.560 *	−0.412	−0.159	**LDL-_C_**
		−0.003	−0.106	0.245	**Triglycerides**
			−0.679 *	−0.003	**Body Weight**
				0.214	**FO_MAX_**

The required coefficient of correlation for 22 subjects at * *p* < 0.05, is r = 0.423.
